# Liver transplantation in patients over 70 years old

**DOI:** 10.1590/0102-67202025000025e1894

**Published:** 2025-08-29

**Authors:** Tércio GENZINI, Marina Guitton RODRIGUES, Thais Natalia de ALMEIDA, Fernanda Ribeiro DANZIERE, Luiz Edmundo Pinto da FONSECA, Marcella Costa GENZINI, Fernando Kruglensky LERNER, Aloysio Ikaro Martins COELHO, Keli Camila Vidal GROCHOSKI, Marcelo PEROSA

**Affiliations:** 1 Hospital Alemao Oswaldo Cruz, Liver Transplant Unit – São Paulo (SP), Brazil.; 2 Hospital Leforte, Liver Transplant Unit – São Paulo (SP), Brazil.; 3 Hospital das Clínicas de Rio Branco, Liver Transplant Unit – Rio Branco (AC), Brazil; 4 Grupo Hepato, Liver Transplant Unit – São Paulo (SP), Brazil.; 5 Santa Casa de São Paulo, Faculdade de Ciências Médicas – São Paulo (SP), Brazil.

**Keywords:** Liver Transplantation, Elderly, Survival, Transplante de Fígado, Idosos, Sobrevida

## Abstract

**Background:**

Liver transplantation (LT) is increasingly recognized as a treatment option for various diseases affecting a growing elderly population. However, its use in patients over 70 years of age remains controversial in centers with suboptimal outcomes or high waitlist mortality.

**Aim:**

The aim of this study was to evaluate the effectiveness of LT as a treatment option for elderly patients aged 70 years or older, in comparison with younger recipients.

**Methods:**

This retrospective study was conducted based on medical record data from 309 liver transplant recipients treated by the same surgical team across three hospitals — two located in São Paulo, São Paulo state (SP) and one in Rio Branco, Acre state (AC). Patients were divided into two groups for comparison: those aged up to 69 years (Group I) and those aged 70 years or older (Group II).

**Results:**

Donor characteristics were similar between the two groups, except for a higher norepinephrine dose in Group I (p<0.05). Group II showed greater transfusion requirements and longer intensive care unit (ICU) stays (p<0.05), as well as higher rates of malnutrition and comorbidities. Notably, 90-day survival was comparable between the groups.

**Conclusions:**

Patients aged 70 years or older can achieve outcomes comparable to those of younger recipients, provided they receive grafts from carefully selected donors. This population should not be excluded from transplant waitlists, and specific allocation policies or scoring adjustments should be considered to ensure equitable access.

## ARTICLE HIGHLIGHTS

- Liver transplantation (LT) in patients aged ≥70 years is feasible with selected donors.
- Short-term outcomes were comparable to those in younger recipients.
- Elderly patients had higher intensive care unit (ICU) stay and transfusion needs.
- Advanced age should not be a contraindication for LT when carefully evaluated.

## CENTRAL MESSAGE

A retrospective analysis of liver transplants was performed, comparing patients over and under 70 years of age. The elderly group was transplanted with careful donor selection and obtained results comparable to those of the younger group.

## PERSPECTIVES

This study aims to show that elderly patients over 70 years of age can have good results after liver transplantation, comparable to patients under 70 years of age, with good donor selection and perhaps additional points to favor their position on the waiting list.

## VISUAL ABSTRACT

**Figure:**
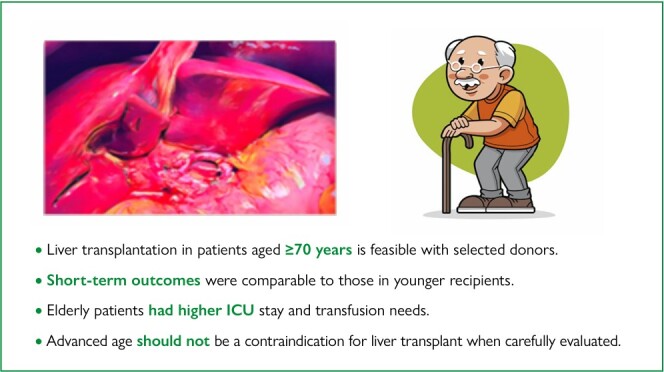

## RESUMO

**Racional:**

O transplante de fígado é cada vez mais reconhecido como uma opção de tratamento para diversas doenças que afetam uma crescente população idosa. No entanto, seu uso em pacientes com mais de 70 anos permanece controverso em centros com menos desenvolvidos ou alta mortalidade em lista de espera.

**Objetivos:**

Avaliar a eficácia do transplante de fígado como opção de tratamento para pacientes idosos com 70 anos ou mais, em comparação com receptores mais jovens.

**Métodos:**

Estudo retrospectivo baseado em dados de prontuários médicos de 309 receptores de transplante de fígado tratados pela mesma equipe cirúrgica em três hospitais — dois localizados em São Paulo (SP) e um em Rio Branco (AC). Os pacientes foram divididos em dois grupos para comparação: aqueles com até 69 anos (Grupo I) e aqueles com 70 anos ou mais (Grupo II).

**Resultados:**

As características dos doadores foram semelhantes entre os dois grupos, exceto por uma dose maior de norepinefrina no Grupo I (p<0,05). O Grupo II apresentou maior necessidade de transfusão e maior tempo de internação na UTI (p<0,05), além de maiores taxas de desnutrição e comorbidades. A sobrevida em noventa dias foi comparável entre os grupos.

**Conclusões:**

Pacientes com 70 anos ou mais podem alcançar evoluções comparáveis aos de receptores mais jovens, desde que recebam enxertos de doadores cuidadosamente selecionados. Essa população não deve ser excluída das listas de espera para transplante, e políticas específicas de alocação ou ajustes de pontuação devem ser considerados para garantir acesso equitativo.

## INTRODUCTION

 Liver transplantation (LT) stands as one of the major advancements in 20th-century medicine, with its indications expanding in parallel with improved outcomes and growing success rates. 

 The aging of the global population and the substantial increase in life expectancy over the last century, from 45–50 years to over 80 years^19^, combined with changes in the epidemiological profile of chronic liver diseases, have led to a growing number of patients over 70 years old being referred for LT. 

 In many transplant centers, the number of recipients over the age of 65 is steadily increasing; however, transplantation in patients over 70 years old remains a subject of ongoing debate^2,20^. 

 Advanced age and its associated comorbidities are recognized risk factors that may negatively impact transplant outcomes, leading to reduced patient and graft survival. This scenario often raises concerns regarding transplant candidacy in the context of organ scarcity, ultimately resulting in more restrictive eligibility criteria for elderly recipients^19^. 

 Although Brazilian legislation permits the inclusion of patients aged 70 and above on the transplant waitlist, many centers still decline these candidates due to the higher morbidity and mortality associated with this age group, as well as concerns about waitlist mortality among younger patients. This decision often reflects a philosophical stance that is not entirely supported by the current literature, although it may be justified by center-specific experiences. 

 The need for this therapeutic alternative in the elderly population is unquestionable, which justifies the present study aimed at reporting the experience of a single surgical team, following a standardized protocol across three different hospitals. 

## METHODS

 This was a retrospective study based on data collected from the electronic medical records of 309 patients who underwent LT performed by the same surgical team at three hospitals — two located in São Paulo, São Paulo state (SP) and one in Rio Branco, Acre state (AC). 

 The objective of this study was to perform a comparative analysis between patients aged up to 69 years and those aged 70 years or older at the time of LT. 

 Data were collected on preoperative clinical characteristics of recipients, donor variables, and clinical outcomes for 309 liver transplant recipients across three healthcare institutions: two in the city of São Paulo (SP) (Hospital Alemão Oswaldo Cruz and Hospital Leforte – DASA) and one in the city of Rio Branco (AC) (Hospital das Clínicas de Rio Branco). 

 All patients and/or their legal guardians signed an informed consent form at the time of their inclusion on the liver transplant waiting list, prior to undergoing transplantation, authorizing the use of their clinical, laboratory, and imaging data for research and scientific publication purposes, provided their identities remained confidential. 

### Statistical analysis

 Descriptive statistics included minimum, maximum, median (P50), interquartile range (P25–P75), mean, and standard deviation (SD), in addition to percentages, to summarize the variables studied. The association or dependence between two categorical variables was assessed using Pearson’s Ꞌ^2^ test. Fisher’s exact test was also applied to evaluate associations between categorical variables, particularly for comparisons of event proportions between groups. This test was recommended due to the small sample size, especially in patients aged >70 years. 

 To compare two independent groups (≤69 vs. ≥70 years) in terms of the mean of an interval variable (e.g., age), the independent samples Student’s t-test was used. 

 Levene’s test was applied to assess the homogeneity of variances for each variable between the two groups. 

 This test aims to determine whether the variances of a given variable differ significantly between groups, with a significance level set at p<0.05. 

 In this study, variance heterogeneity was assumed; therefore, results from the Student’s t-test were interpreted under the assumption of unequal variances, providing more robust statistical inferences. 

 All results were considered statistically significant when the p-value was less than 0.05, corresponding to a confidence level of at least 95%. 

## RESULTS

 A total of 309 patients were included in this study: 213 from São Paulo state (SP) and 96 from Acre state (AC). 

 LTs were performed over the past 5 years in São Paulo city (SP) and since the beginning of the transplant program in Rio Branco (AC), which started in 2014. These timeframes were defined based on the availability and completeness of clinical data from donors and recipients at each institution. Records prior to the analyzed period were either incomplete or inaccessible in the electronic medical charts, making their inclusion in the analysis unfeasible. 

 Regarding recipient age, 292 patients were between 14 and 69 years old (Group I), while 17 patients were between 70 and 78 years old (Group II). 

Table 1 presents the preoperative characteristics of the liver transplant recipients.As shown, in patients aged 70 years or older (Group II), the majority of patients (64.7%) had blood type A. In contrast, among patients aged up to 69 years (Group I), 41.8% had blood type A and 39% had blood type O. No statistically significant differences were observed between the two age groups (p>0.05). 

**Table 1:** Preoperative characteristics of liver transplant recipients by age group (n=309).

Characteristics		Group I (≤69 years)	Group II (≥70 years)	p-value
Blood type (%)				
	A	122 (41.8)	11 (64,7)	0.227*
	AB	19 (6,5)	1 (5,9)	
	B	37 (12,7)	2 (11,8)	
	O	114 (39,0)	3 (17,6)	
Liver function (MELD score)				
	Minimum–Maximum	7,0–56,0	8,0–31,0	0.138†
	Mean±SD	22,1±8,8	19,5±6,4	
	Median (P25–P75)	21.0 (15.0; 29.0)	19.0 (15.0; 23.0)	
Liver function (categories) (%)				
	≤20	165 (56.5)	14 (82.4)	0.336*
	21–24	35 (12.0)	0 (0.0)	
	25–29	34 (11.6)	1 (5.9)	
	30–35	37 (12.7)	2 (11.8)	
	>35	21 (7.2)	0 (0.0)	
Child-Pugh classification (%)				
	Class A	72 (24.7)	6 (35.3)	0.356*
	Class B	143 (49.1)	9 (52.9)	
	Class C	76 (26.1)	2 (11.8)	
Nutritional status (%)				
	Eutrophic	159 (54.6)	7 (41.2)	0.146*
	Mild malnutrition	47 (16.2)	6 (35.3)	
	Moderate malnutrition	37 (12.7)	3 (17.6)	
	Severe malnutrition	48 (16.5)	1 (5.9)	
Body mass index (BMI, kg/m ^²^)				
	Minimum–Maximum	14.0–43.9	20.8–33.3	0.378†
	Mean±SD	25.9±4.4	26.7±3.6	
	Median (P25–P75)	25.2 (23.0; 27.9)	26.6 (23.5; 29.7)	

MELD: Model for End-Stage Liver Disease; BMI: Body mass index; SD: Standard deviation.

* Ꞌ^2^ test;

† student’s t-test for independent samples.

 Regarding liver function assessed by the Model for EndStage Liver Disease (MELD) score, values ranged from 7 to 56 in Group I, with a mean of 22.1, and from 8 to 31 in Group II, with a mean of 19.5. When classifying liver function severity, the majority of patients in both age groups had an MELD score of ≤20 (56.5% in Group I and 82.4% in Group II). As for the Child-Pugh classification, most patients were categorized as Class B (49.1% in Group I and 52.9% in Group II). No statistically significant differences were observed between the age groups (p>0.05) (Table 1). 

 No significant differences were observed between the two age groups in terms of nutritional status or body mass index (BMI) (p>0.05). In Group I, 54.6% of the patients were classified as eutrophic, whereas in Group II, 41.2% were eutrophic and 35.3% presented with mild malnutrition. BMI in Group I ranged from 14 to 43.9 kg/m ^2^, with a mean of 25.9 kg/m^2^, while in Group II, it ranged from 20.8 to 33.3 kg/m^2^, with a mean of 26.7 kg/m2. In both groups, the majority of patients had a BMI below 30 kg/m2, indicating the absence of obesity (Table 1). 

 Approximately 60% of the patients in Group I and 70.6% in Group II were classified under Indication Group A for transplantation (including hepatocellular carcinoma [HCC], polycystic liver disease, metabolic disorders, disabling encephalopathy, intractable pruritus, viral hepatitis, and hemochromatosis), which was considered less severe by the transplant team. Additionally, 28.2% of the patients in Group I and 17.6% in Group II fell into Indication Group B (including combined liver-kidney transplantation, nonalcoholic steatohepatitis [NASH], and alcohol-related cirrhosis), which was considered more severe. No statistically significant differences were observed between the two groups (p> 0.05) (Table 2). 

**Table 2:** Preoperative patient characteristics by recipient age group (n=309).

Characteristics		Group I (≤69 years)	Group II (≥70 years)	p-value
BMI (categories) (%)				
	<30	257 (88.0)	14 (82.4)	0.512
	30–35	23 (7.9)	3 (17.6)	
	35.1–40	7 (2.4)	0 (0.0)	
	>40	5 (1.7)	0 (0.0)	
Transplant indication (%)				
	Group A	168 (57.7)	12 (70.6)	0.686
	Group B	82 (28.2)	3 (17.6)	
	Group C	36 (12.4)	2 (11.8)	
	Group D	5 (1.7)	0 (0.0)	
Previous surgeries (%)				
	No	235 (80.5)	15 (88.2)	0.750
	Yes	57 (19.5)	2 (11.8)	
Preoperative comorbidities and conditions (%)				
	Group A	172 (58.9)	5 (29.4)	<0.001
	Group B	25 (8.6)	8 (47.1)	
	Group C	23 (7.9)	3 (17.6)	
	Group D	40 (13.7)	1 (5.9)	
	Group E	32 (11.0)	0 (0.0)	

p-values refer to the Fisher’s exact test. BMI: Body mass index.

 The criteria for clinical severity classification were established based on the underlying etiology of liver disease, according to the medical team’s experience. Patients with conditions typically associated with slower progression or more favorable prognosis (e.g., HCC and controlled viral hepatitis) were considered less severe and allocated to Group A. In contrast, those with diseases associated with higher clinical risk, need for combined organ transplantation, or severe complications (e.g., alcohol-related cirrhosis, NASH, and refractory ascites) were categorized into higher severity groups (Groups B–D). 

 This categorization was developed exclusively for statistical analysis purposes and does not reflect universally standardized criteria. Rather, it represents a functional classification adapted to the clinical practice realities of the participating centers. 

 In Group I, 19.5% of the patients had a history of previous surgeries, compared to 11.8% in Group II. No statistically significant difference was observed between the groups (p> 0.05) (Table 2). 

 Regarding comorbidities and preoperative clinical conditions, a statistically significant difference was found between the two age groups (p<0.05). In Group I, the majority of patients (58.9%) had no documented comorbidities, whereas in Group II, 47.1% of the patients had at least one comorbidity—most commonly insulin-dependent diabetes and/or hypertension (Table 2). 

Table 3 presents the characteristics of the donors. As shown, statistically significant differences (p<0.05) were found only in relation to the norepinephrine dosage, which was significantly higher among donors for Group I compared to those for Group II. For all other donor-related parameters—including age, BMI, length of ICU stay, presence of positive cultures, elevated serum sodium, and higher levels of alanine aminotransferase (ALT), gamma-glutamyl transferase (GGT), total bilirubin (TB), and direct bilirubin (DB) — no statistically significant differences were observed between Groups I and II (Table 4). 

**Table 3:** Donor characteristics by recipient age group (n=309).

Donor characteristics		Group I (≤69 years)	Group II (≥70 years)	p-value
Age				
	Minimum–maximum	5.0–71.0	11.0–70.0	0.589*
	Mean±SD	39.2±15.4	41.7±18.1	
	Median (P25–P75)	40.0 (24.0; 51.0)	45.0 (25.0; 56.0)	
Body mass index (BMI, kg/m ^²^)				
	Minimum–maximum	14.0–54.0	17.0–38.0	0.637*
	Mean±SD	26.1±5.2	25.5±4.6	
	Median (P25–P75)	25.0 (23.0; 29.0)	25.0 (23.0; 27.5)	
ICU stay (days)				
	Minimum–maximum	0.0–23.0	1.0–11.0	0.659*
	Mean±SD	5.5±3.4	5.2±2.5	
	Median (P25–P75)	5.0 (3.0; 7.0)	4.5 (4.0; 6.8)	
Norepinephrine dose (μg/kg/min)				
	Minimum–maximum	0.00–1.22	0.00–0.31	0.045*
	Mean±SD	0.16±0.19	0.11±0.09	
	Median (P25–P75)	0.11 (0.02; 0.21)	0.10 (0.03; 0.18)	
Positive culture (%)				
	No	265 (92.7)	17 (100.0)	0.618†
	Yes	21 (7.3)	0 (0.0)	
Highest sodium level (mEq/L)				
	Minimum–Maximum	125.0–200.0	139.0–177.0	0.809*
	Mean±SD	152.6±12.9	153.2±10.1	
	Median (P25–P75)	150.0 (143.0; 160.0)	153.0 (145.0; 158.0)	

SD: Standard deviation; BMI: Body mass index; ICU: Intensive care unit.

* Student’s t-test for independent samples

† Fisher’s exact test.

**Table 4:** Donor liver function parameters by recipient age group (n=309).

Donor characteristics		Group I (≤69 years)	Group II (≥70 years)	p-value
Highest ALT (U/L)				
	Minimum–maximum	5.0–2083.0	9.0–430.0	0.338*
	Mean±SD	94.4±188.0	68.8±97.5	
	Median (P25–P75)	47.0 (28.0; 84.0)	33.0 (22.5; 75.5)	
Highest GGT (U/L)				
	Minimum–maximum	7.0–965.0	15.0–370.0	0.281*
	Mean±SD	103.6±136.0	78.6±87.1	
	Median (P25–P75)	55.0 (26.0; 119.0)	48.0 (26.5; 105.0)	
Highest total bilirubin (mg/dL)				
	Minimum–maximum	0.00–6.00	0.00–4.17	0.908*
	Mean±SD	0.79±0.88	0.83±1.21	
	Median (P25–P75)	0.66 (0.00; 1.00)	0.39 (0.00; 1.00)	
Highest direct bilirubin (mg/dL)				
	Minimum–maximum	0.00–3.43	0.00–2.46	0.670*
	Mean±SD	0.36±0.60	0.29±0.62	
	Median (P25–P75)	0.10 (0.00; 0.50)	0.00 (0.00; 0.35)	

ALT: Alanine aminotransferase; GGT: Gamma-glutamyl transferase; SD: Standard deviation.

* Student’s t-test for independent samples.

 Complete data on cold ischemia time (i.e., the interval between organ procurement and implantation) were not consistently recorded in the electronic medical records, preventing their inclusion in the statistical analysis. 

Table 5 summarizes the clinical outcomes assessed in this study. A statistically significant difference (p<0.05) was observed regarding the number of blood transfusion units required, with Group II presenting a significantly higher transfusion requirement compared to Group I. For the other outcomes — including length of ICU stay, total hospital stay, 90-day post-transplant mortality, need for dialysis, and 1-year post-transplant survival, no statistically significant differences were identified at the 5% significance level. However, at an 8% significance threshold, differences between the two age groups were noted regarding 90-day mortality and 1-year survival: Group I showed a higher rate of death within 90 days and a lower 1-year survival rate compared to Group II. 

**Table 5:** Clinical outcomes by recipient age group (n=309).

Outcome		Group I (≤69 years)	Group II (≥70 years)	p-value
Transfusion units				
	Minimum–maximum	0.0–14.0	0.0–7.0	0.009*
	Mean±SD	1.6±2.4	3.1±2.1	
	Median (P25–P75)	0.0 (0.0; 2.0)	3.0 (1.5; 4.5)	
ICU stay (days)				
	Minimum–maximum	0.0–190.0	2–37	0.192*
	Mean±SD	6.5±14.4	9.9±9.4	
	Median (P25–P75)	3.0 (2.0; 6.0)	7.0 (4.0; 9.8)	
Hospital stay (days)				
	Minimum–maximum	0.0–190.0	7.0–37.0	0.831*
	Mean±SD	16.0±21.6	16.6±9.0	
	Median (P25–P75)	10.0 (7.0; 16.0)	15.0 (9.0; 23.0)	
90-day mortality				
	No	250 (85.6%)	17 (100.0%)	0.078†
	Yes	42 (14.4%)	0 (0.0%)	
Dialysis				
	No	270 (92.5%)	15 (88.2%)	0.631†
	Yes	22 (7.5%)	2 (11.8%)	
1-year survival				
	No	63 (22.9%)	0 (0.0%)	0.079†
	Yes	212 (77.1%)	13 (100.0%)	

* Student’s t-test

† Fisher’s exact test.

SD: Standard deviation; ICU: Intensive care unit.

 In the preoperative comparison, liver function, assessed by Child-Pugh classification and MELD-Na+ score, was similar between Groups I and II (MELD-Na+: 19.6 vs. 22.4, p=0.69, p>0.5; Child-Pugh A: 26.6 vs. 26.3%, B: 46.6 vs. 41.1%, and C: 26.6 vs. 32.5%, p=0.45, p>0.5). However, the degree of malnutrition was higher in Group II, with 40% of cases classified as having severe malnutrition (p<0.05). In Group II, 8 patients (53.3%) were listed as special cases due to HCC, 2 (13.3%) due to refractory ascites, 1 (6.7%) due to hepatic encephalopathy (HE), and 1 (6.7%) due to hepatic hydrothorax. During hospitalization, Group II had a significantly longer ICU stay (5 vs. 3 days, p<0.05), while mechanical ventilation time was similar between groups (1 vs. 1 day, p=0.76, p>0.05). Hemodialysis was more frequent in Group I, although the difference was not statistically significant (0 vs. 21.3%, p=0.32, p>0.5). The overall length of hospital stay was comparable between the groups (9.5 vs. 8 days, p=0.51, p>0.05). 

 Early survival (within 90 days post-transplant) was also similar between Groups I and II (86.9 vs. 90%, p=1.0, p>0.5). 

### Classification of clinical severity used in this study

 Transplant indication groups (based on clinical severity as defined by the medical team).

- Group A – Low severity: HCC, polycystic liver disease, disabling encephalopathy, intractable pruritus, viral hepatitis, and hemochromatosis.
- Group B – Moderate severity: combined liver–kidney transplantation, metabolic-associated (MASH), and alcohol-related cirrhosis.
- Group C – High severity: refractory ascites, hepatic hydrothorax, retransplantation, and Budd-Chiari syndrome.
- Group D – Highest severity: fulminant hepatitis.

Preoperative comorbidities and clinical conditions (stratified by severity).

- Group A – No comorbidities.
- Group B – Insulin-dependent diabetes mellitus and systemic arterial hypertension.
- Group C – Partial portal vein thrombosis, renal insufficiency (creatinine clearance between 30 and 50 mL/min), prior dialysis, and anticoagulant use.
- Group D – Complete portal vein thrombosis, ongoing dialysis or creatinine clearance <30 mL/min, mechanical ventilation, and hospitalization in the general ward.
- Group E – Use of vasoactive drugs (VADs), depressed level of consciousness, and admission to the intensive care unit (ICU).

## DISCUSSION

 LT in patients over 70 years of age remains a complex and challenging issue, primarily due to factors such as poorer outcomes and the ethical implications of increased waitlist mortality among younger candidates. Current studies suggest that overall post-transplant survival is higher in younger recipients, with a mean age of approximately 40 years, based on data from the United Kingdom and the United States. 

 Gil et al. compared liver transplant outcomes between middle-aged recipients and those aged over 70 and found that the risk of mortality in the elderly group was approximately four times higher after adjusting for underlying liver disease (odds ratio [OR] 4.1; 95% confidence interval [CI] 2.217.58) and nearly three times higher after adjusting for both liver disease and perioperative complications (OR 2.92; 95%CI 1.37–6.24)^12^. Furthermore, the cost of LT was shown to increase significantly with advancing age, reinforcing the need for cautious consideration when selecting elderly recipients^12^. 

 Moreover, the systematic review and meta-analysis conducted by Charlton et al. concluded that increased recipient age was significantly associated with higher post-transplant mortality (hazard ratio [HR] 2.07; 95%CI 1.71–2.50; p=0.40, p>0.5)^5^. 

 However, a shift in the etiological profile of liver transplant candidates, particularly the rising prevalence of metabolismrelated liver diseases, has led to an increase in the age of transplant recipients. As a result, LT in elderly patients is becoming an increasingly necessary therapeutic option. 

 In 1988, only 1.7% of liver transplants performed in the United States were in recipients over the age of 65. By 2016, this percentage had risen to 18.7%^2,15^, and in 2017, to 20%^10^. These changes have been attributed to an increase in the average age at the time of waitlist registration, as well as to shifts in the etiological profile of liver diseases leading to transplantation. There has been a marked rise in conditions associated with metabolic syndrome, such as nonalcoholic fatty liver disease (NAFLD) and HCC, along with a decline in viral hepatitis-related indications^10^. 

 In the cases analyzed by our team, this etiological profile was clearly evident (Table 2), with a higher incidence of MASH among patients aged 70 years or older (Group II). 

 Several authors have reported LT outcomes in elderly patients, with some studies showing poor results and others reporting favorable outcomes. There is increasing recognition that chronological age should not be considered an isolated criterion, and that physiological age may represent a more meaningful indicator of transplant eligibility^19^. 

 Cross et al. compared liver transplant outcomes among recipients aged 60–64 years, ≥65 years, and <60 years, and found similar graft and patient survival rates at 30 days, 1 year, and 5 years, respectively^9^. 

 However, in patients aged >65 years, MELD scores were lower, hepatitis C was less common, and the proportion of patients with primary biliary cholangitis was higher. 

 Lipshutz et al. also demonstrated comparable outcomes after LT in septuagenarians versus younger patients, provided that physiological age and pretransplant clinical conditions were thoroughly assessed^14^. 

 Studies such as those by Kim et al.^13^ and Freitas et al.^11^ emphasize that elderly patients can achieve satisfactory outcomes after LT, provided they are carefully selected based on a comprehensive evaluation of their clinical condition and their ability to tolerate both the surgical procedure and the postoperative rehabilitation process. 

 Croome et al. compared simultaneous liver–kidney transplantation in patients over 65 years of age (n=8,495) with those under 65 years of age (n=4,517) and observed similar patient survival outcomes between the two groups^8^. It is important to emphasize that the decision to perform LT in elderly patients must be based on an individualized approach, carefully weighing the risks and benefits of the procedure for each specific case. 

 Beyond physiological reserve — which is influenced by factors such as physical activity, diet, smoking, alcohol use, and others—psychosocial comorbidities such as depression can significantly impact clinical outcomes after transplantation. A meta-analysis of 27 studies involving over 1,000 patients demonstrated that post-transplant depression was associated with a 65% increased risk of both mortality and graft loss^15^. 

 In the analyses by Chen et al. in Taiwan, increased mortality was observed from the age of 60 onward^6^, while Sony et al. in the United States reported similar findings^18^. 

 However, both studies demonstrated that cardiac and renal comorbidities, HE, thrombocytopenia, TB >3.5 mg/dL, and serum albumin <2.65 mg/dL were associated with worse outcomes^6,18^. 

 These studies also showed that pretransplant BMI and smoking history further increased post-transplant mortality in elderly recipients. 

 Slattery et al. compared LT outcomes in patients over 65 years of age (n=40) versus those under 65 years of age (n=511) using data from the Irish national registry and observed lower survival rates in the older group — 77.8 vs. 93% at 1 year and 64.5 vs. 85% at 3 years^17^. 

 Schwartz et al. compared LT outcomes in patients with HCC, aged >70 versus < 70 years, and found lower survival rates in the older group, both at 1 year (81.1 vs. 88.4%) and at 5 years (55.2 vs. 72.7%)^16^. 

 Aduen et al., from the Mayo Clinic, reported similar outcomes when comparing 42 liver transplants performed in patients over 70 years of age with 42 transplants in patients under 60 years of age^1^. 

 Conversely, Collins et al., from Wisconsin, observed lower survival rates at 5 years (52 vs. 75%) and 10 years (35 vs. 60%) when comparing 91 transplants in patients over 60 years of age with 387 transplants in those under 60 years of age^7^. 

 In comparative studies, elderly recipients often present with lower BMI, absence or well-controlled diabetes mellitus, lower international normalized ratio (INR), and higher serum albumin levels at the time of waitlist registration. 

 These factors contribute to lower MELD scores and reduced clinical severity, which may partially explain the more favorable outcomes reported in several series^8,9,11,13,14^. 

 Indeed, nutritional status and the degree of sarcopenia are critical components of preoperative assessments. However, in addition to age-related protein-energy malnutrition and sarcopenia, elderly patients often exhibit slower responses to nutritional therapies. Furthermore, logistical constraints related to transplant waitlists frequently hinder the timely and adequate implementation of such interventions. In this study, most patients in Group II were classified as having mild-to-moderate malnutrition based on the Subjective Global Assessment (SGA), while a higher incidence of severe malnutrition was observed in Group I. However, no statistically significant difference was found between the two groups (Table 1). 

 In the current context, it is not feasible to selectively stratify clinical severity based on advanced age. In the city of São Paulo (SP), the average MELD scores at the time of transplantation for the most common blood groups (O and A) are 29. Since no additional points are assigned to account for the increased risk associated with advanced age^4^, elderly patients evaluated by our team competed equally with younger candidates, based solely on MELD severity and, in some cases, on special exception points granted for refractory ascites, HCC, or hepatic hydrothorax (Table 2). 

 Elderly patients tolerate shorter waiting periods and present higher mortality and removal rates from the waitlist due to clinical deterioration. This results in a lower likelihood of undergoing transplantation and a higher incidence of posttransplant complications. Patients over 70 years of age tend to die with lower MELD scores, have higher dropout rates, and are less likely to reach higher MELD thresholds or undergo transplantation^10^. 

 Early mortality in elderly patients undergoing LT increases significantly when the MELD score exceeds 256. Another factor that complicates transplantation in older recipients is the inability to use extended criteria donors, due to the high postoperative mortality associated with these grafts^3^. 

 Following the team’s routine practice of carefully matching donors and recipients, and considering age >70 years as a relevant indicator of clinical severity, donors are selected based on lower vasopressor requirements and reduced expected cold ischemia times in transplants performed in this subgroup (Table 3). 

 In 2010, Aloia et al. published a study demonstrating an association between the sum of donor and recipient ages and post-transplant outcomes, showing worse results when this combined age was ≥120^2^. Although this specific criterion is not applied by the team evaluated in this study, efforts are made to limit donor age to under 50 years, maintain a donor risk index (DRI) below 1.4, use low-dose vasopressors, and ensure cold ischemia times of <8 h. 

 In addition, the higher prevalence of depression among elderly individuals requiring medical treatment is well documented in the literature. However, this factor is rarely analyzed in the context of transplant waitlists or postoperative outcomes in this population. 

 Among the patients evaluated in this study, depression and loss of appetite were frequently observed, affecting approximately 20% of cases, and often necessitating enteral nutrition via a nasoenteric tube. At the center evaluated, it is a routine practice to place and position the nasoenteric feeding tube at the end of the surgical procedure, prior to abdominal closure. 

 Although the risks associated with advanced age and its related comorbidities are well recognized, and despite the ongoing debate regarding the potential impact of including elderly patients on waitlists on the mortality of younger candidates, the favorable outcomes observed in this study support the continued offering of LT to patients over 70 years of age. 

 In this cohort, elderly recipients required a greater volume of transfusion due to higher cardiac risk and experienced longer ICU stays. Interestingly, they had a lower incidence of dialysis and achieved survival outcomes comparable to their younger counterparts (Table 5). 

 Moreover, an increasing number of meaningful contributions to society are made by older individuals, which should, from this perspective, justify the assignment of additional priority points on transplant waitlists and preferential allocation of younger and hemodynamically stable donors for elderly recipients. 

 The sample size of patients aged 70 years or older remains small, which limits the statistical power for certain types of analysis. Furthermore, donor selection was more rigorous in the elderly recipient group, which, although not statistically significant in the present analysis, introduces a potential bias in outcome interpretation. 

## CONCLUSIONS

 LT in patients aged 70 years or older yields outcomes comparable to those observed in younger recipients, provided that grafts are obtained from carefully selected donors meeting more stringent criteria. 

 As with pediatric candidates, advanced age should be considered an additional allocation factor, warranting priority points for patients aged 70 years and above on liver transplant waitlists. 

## Data Availability

The information regarding the investigation, methodology, and data analysis of the article is archived under the responsibility of the authors.
